# Probiotic Kefir Improves Renal Disorders in Ovariectomized Female SHR with High Fructose Intake–Induced Metabolic Syndrome

**DOI:** 10.1007/s12602-025-10490-w

**Published:** 2025-03-13

**Authors:** Leonardo da Silva Escouto, Thatiany Jardim Batista, Pollyana Peixoto, Felipe Tonon Firmino, Silas Nascimento Ronchi, Maria Eduarda de Souza Barroso, Edgar Hell Kampke, Tadeu Uggere de Andrade, Antonio Ferreira de Melo Junior, Nazaré Souza Bissoli

**Affiliations:** 1 Department of Physiological Sciences, Federal University of Espírito Santo, Vitória, Espírito Santo Brazil; 2 Department of Pharmacy, University Vila Velha (UVV), Vila Velha, Brazil; 3 iNOVA4HEALTH, NOVA Medical School, Faculdade de Ciências Médicas (NMS/FCM), Universidade Nova de Lisboa, 1159-056 Lisbon, Portugal; 4 Centro Clínico e Académico de Lisboa, 1156-056 Lisbon, Portugal

**Keywords:** Kefir, Renal function, Metabolic syndrome, Female SHR, Oxidative stress

## Abstract

Women in postmenopausal period may present several comorbidities linked to metabolic syndrome (MetS). Our hypothesis is that kefir may prevent the deleterious effects in renal function in an experimental model of metabolic syndrome (MetS) and ovarian hormone deficiency. Young female spontaneously hypertensive rats (SHR) were divided into four groups: ovariectomized (OVX) control, OVX fructose, OVX kefir, and OVX kefir + fructose. They received kefir (5% w/v) via gavage for 8 weeks, while fructose (10% w/v) was available ad libitum. In ponderal parameters and glucose metabolism, we observe that fructose-overloaded groups (OF and OKF) showed increased weight, visceral fat, and fasting blood glucose. However, OKF partially reduced glycemic peak in the glucose tolerance test. Moreover, the standard method for the measurement of renal function showed that OF and OKF groups had a reduction in glomerular filtration rate, and surprisingly OKF exhibited increased renal flow (RBF and RPF) and decreased resistance (RVR). These might be associated with the findings in oxidative stress and nitric oxide (NO) bioavailability, in which kefir in the OKF group was capable of increasing total nitrogen oxides (NOx), attenuate the generation of hydrogen peroxide (DCF) and peroxynitrite (HPF), and also decreased the elevated microalbuminuria promoted by fructose even though the systemic blood pressure between the groups did not differ. Taking together our results, in the present study, kefir showed favorable effects in the model of metabolic syndrome and ovarian hormone deficiency (OKF), potentially protecting the kidney from the deleterious effects of fructose.

## Introduction

Arterial hypertension affects more than a quarter of the world's population, being a significant risk factor for cardiovascular and renal diseases [1–4]. Hypertension can be worsened by comorbidities such as dyslipidemia, obesity, glucose intolerance, and diabetes mellitus [5]. Additionally, metabolic syndrome (MetS) is a multifactorial disease, and it is also linked to the development of renal disease [6–8], with potential mechanisms including endothelial and mitochondrial dysfunction, inflammation, oxidative stress, insulin resistance, and hyperuricemia [9, 10].

On the other hand, regarding a sugar-rich diet, such as the fructose used in this study, there is a possibility of contributing to the development of insulin resistance [11], dyslipidemia [12], hyperuricemia [13], endothelial dysfunction [14], increased blood pressure, sympathetic system modulation [15], as well as non-alcoholic fatty liver disease. With all these factors related to the high consumption of fructose, they can contribute to the development of MetS.

It is important to note that, in addition to factors such as hypertension and MetS, estrogen deficiency in post-menopausal women is also involved in the rapid progression of the aforementioned comorbidities [10, 16, 17]. Additionally, a study by Melo Junior et al. found that female ovariectomized (OVX) spontaneously hypertensive rats (SHR) presented an exacerbation of glomerular lesion severity. Although the mechanisms involved are not yet fully understood, it can be said that sexual dimorphism in the progression of chronic kidney disease and acute kidney injury are mainly mediated by sex hormones, with the protection found in women attributed to the protective effects of ovarian hormones [18–21].

However, the proposal of hormone replacement therapy in the post-menopausal phase is a controversial topic, with many studies pointing to an increased health risk [22–24]. Therefore, alternative therapies that can prevent and/or control cardiovascular and renal changes that occur in the post-menopausal phase, and that do not lead to problems related to classical hormone therapy, are of great interest in studies [25–27]. Thus, the probiotic kefir has become an object of interest in our study as a non-pharmacological tool.

According to the Food and Agriculture Organization of the United Nations and the World Health Organization, probiotics are defined as live microorganisms that, when administered in sufficient amounts, provide health benefits to the host [28].

Kefir is a fermented milk beverage with an acidic character and low alcohol content, produced through the fermentation of kefir grains containing several bacteria and yeasts. These microorganisms exist in a complex symbiotic relationship and contribute to the health benefits of kefir, often classified as a probiotic [29–31]. However, while kefir contains microorganisms with probiotic properties and biological activities, its composition can vary depending on production conditions, differing in their characteristics and influencing the consistency of the probiotic strains present, such as kefir's exopolysaccharide (kefiran) or being prepared in milk or water. Therefore, it is more accurate to classify kefir as a product with probiotic action rather than a standardized probiotic product [32–34].

Although kefir originated in the Balkans of Eastern Europe, its use has spread across the globe over time, largely due to its numerous health benefits [35–41]. Today, kefir holds a significant position in the global fermented beverage market, with its market size valued at USD 1.23 billion in 2019 and projected to reach USD 2.40 billion by 2032 [42]. Additionally, the widespread practice of homemade kefir production makes it challenging to accurately measure its consumption statistics [36, 43].

This probiotic has been used as a study tool in both experimental and clinical models for hypertension, diabetes, and MetS-related comorbidities, among others [44–46]. However, the literature still needs results in the experimental model of female rats, SHR, OVX, and subjected to high fructose intake to achieve a MetS model. Considering the previous beneficial effects of kefir, we hypothesized that the treatment with this probiotic may prevent renal deleterious effects of ovarian deficiency and high fructose intake in female SHR. This study aimed to evaluate the effect of kefir treatment on renal parameters in an experimental model of metabolic syndrome and ovarian hormone deficiency.

## Methods

### Animals

Eight-week-old female SHR with an approximate weight of 130 g were provided by the animal care facility of the Federal University of Espírito Santo (UFES). They were maintained in controlled temperature (20–24 °C) with artificial light–dark cycle (12–12 h) conditions, receiving normal chow diet (NUVILAB CR-1, QUIMTIA S/A) and drinking water ad libitum (Table 1). The animals were randomly divided into four experimental groups (*n* = 7 for each group): OVX control (O); OVX fructose (OF); OVX kefir (OK); OVX kefir + fructose (OKF). This project was approved by our local Ethics Committee in Animal Research (CEUA-UFES) under protocol no. 11/2019, following the appropriate recommendations and guidelines of CEUA-UFES and Conselho Nacional de Controle de Experimentação Animal (CONCEA) [47]. In addition, we estimated a sample size of eight experimental units per group, based on a biologically relevant effect size-to-standard deviation ratio for biochemical analysis, using a power of 90% and a significance level of 5%.

**Table 1:** Ingredient composition and energy intake of the normal chow diet

Composition	
Ground whole corn, soybean meal, wheat bran, calcium carbonate, dicalcium phosphate, sodium chloride (common salt), vitamin A, vitamin D_3_, vitamin E, vitamin K_3_, vitamin B_1_, vitamin B_2_, vitamin B_6_, vitamin B_12_, niacin, calcium pantothenate, folic acid, biotin, choline chloride, iron sulfate, manganese oxide, zinc oxide, copper sulfate, calcium iodate, sodium selenite, cobalt sulfate, lysine, methionine, BHT	
Guaranteed analysis per kilogram of product (g/kg)	
Crude protein	220
Ether extract	50
Crude fiber	70
Vitamins	0.109
Minerals	21.64
Energy (%)	
Carbohydrate	63.4
Protein	25.9
Lipid	10.6

Supplementation per kilogram. Vitamin A (min.) 13,000 IU/kg; vitamin D_3_ (min.) 2000 IU/kg; vitamin E (min.) 34 IU/kg; vitamin K_3_ (min.) 3 mg/kg; vitamin B_1_ (min.) 5 mg/kg; vitamin B_2_ (min.) 6 mg/kg; vitamin B_6_ (min.) 7 mg/kg; vitamin B_12_ (min.) 22 µg/kg; niacin (min.) 60 mg/kg; calcium pantothenate (min.) 21 mg/kg; folic acid (min.) 1 mg/kg; biotin (min.) 0.05 mg/kg; choline (min.) 1900 mg/kg; sodium (min.) 2700 mg/kg; iron (min.) 50 mg/kg; manganese 60 mg/kg; zinc (min.) 60 mg/kg; copper (min.) 10 mg/kg; iodine (min.) 2 mg/kg; selenium (min.) 0.05 mg/kg; cobalt (min.) 1.5 mg/kg; fluorine (max.) 60 mg/kg; lysine (min.) 12 g/kg; methionine (min.) 4000 mg/kg; BHT 100 mg/kg

### Ovariectomy

The animals were anesthetized with ketamine (70 mg/kg; AGENER, BRAZIL) and xylazine (10 mg/kg; BAYER, BRAZIL). We performed a bilateral incision of 1 to 1.5 cm in the skin between the last rib and the thigh, followed by an incision in the muscle layer. The peritoneal cavity was opened for the subsequent removal of the ovaries and ligature of the uterine tube. After the ovaries were removed, the muscle and skin were sutured. The same process was performed on the opposite side, and the animals were relocated to individual cages.

### Fructose Overloading

Animals of OF and OKF groups were subjected to high fructose intake (10% w/v), which was diluted in drinking water ad libitum for 57 days. In the last 3 days of treatment, these groups started receiving only water (washout), so that fructose would not interfere directly with renal function, and anthrone dosage, which is also a reducing sugar. The washout was also followed to metabolic cage analysis.

### Kefir Treatment

**Fig. 1:**
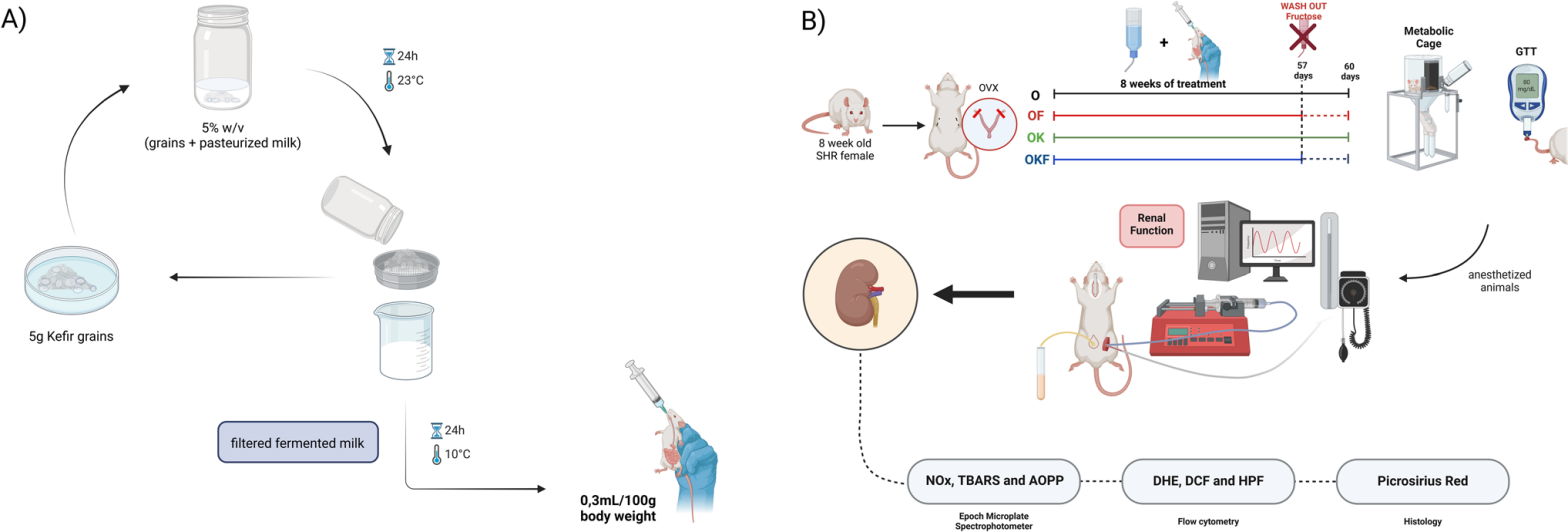
Flowchart of the methodology. (**A**) Kefir preparation. Kefir grains (5% w/v) were added to pasteurized whole milk (Fiore, Santa Teresa, ES, Brazil) and kept at room temperature (20–23 °C) for 24 h. After that, this mixture is filtered, and the resulting product is refrigerated (~ 10 °C) for another 24 h. This procedure was performed daily, where the groups OK and OKF received a daily dose of 0.3 mL/100 g of body weight, and for the respective control groups O and OF, the same dose was administered with the vehicle (milk). (**B**) Experimental design. After undergoing ovariectomy surgery, female SHR were divided into four groups: control (O), OVX fructose (OF), OVX kefir (OK), and OVX kefir + fructose (OKF). Following a 60-day treatment period, the animals were submitted to metabolic cage and a glucose tolerance test (GTT). Renal function was evaluated through an experimental protocol. Biochemical and morphological analyses were performed on kidney tissue, including assessments of total nitrogen oxides (NOx), advanced oxidation protein products (AOPP), thiobarbituric acid reactive substances (TBARS), dihydroethidine (DHE), dichlorofluorescein (DCF), and hydroxyphenyl fluorescein (HPF), in addition to other analyses that were also carried out

The kefir was prepared in the Laboratory of Experimental Hypertension using initial grains obtained from Dr. Celia L. Ferreira at the Federal University of Viçosa, Brazil. In summary, kefir grains (5% w/v) were added to pasteurized whole milk (Fiore, Santa Teresa, ES, Brazil) and kept at room temperature (20–23 °C) for 24 h for the expansion and growth of bacteria and yeasts. After 24 h, this mixture is filtered, and the resulting product is refrigerated (~ 10 °C) to allow the growth of yeasts for another 24 h. This procedure was performed daily, where the groups treated with kefir received a daily dose of 0.3 mL/100 g of body weight, and for the respective control groups O and OF, the same dose was administered with the vehicle (milk). All groups were treated by gavage for a period of 8 weeks (Fig. 1A).

The microbiological analysis of random samples of the grains used showed that the dominant microflora of kefir included various beneficial bacteria (*Acetobacter aceti*, *Acetobacter* sp., *Lactobacillus delbrueckii delbrueckii*, *Lactobacillus fermentum*, *Lactobacillus fructivorans*, *Enterococcus faecium*, *Leuconostoc* spp.), in addition to *Lactobacillus kefiranofaciens*, and yeasts (*Candida famata*, *Candida krusei*). Moreover, the microbiological analysis of kefir grains revealed that the global counting of microorganisms was 7.5 × 107 CFU/mL.

### Animal and Tissue Weights

The animals were weighed weekly during the follow-up. After functional experiments, animals were euthanized and the visceral fat, liver, left kidney, and wet uterus were weighed separately. Additionally, ∆ weight (initial body weight − final) and the organ weight ratio (g) to tibia length (cm) were calculated.

### Fasting Glucose and Glucose Tolerance Test

Fasting glucose assessment and glucose tolerance test (GTT) were performed as described elsewhere [48]. For all tests, blood samples were collected from the tail and glucose levels were measured by a strip-based glucometer (On Call Plus II; Johnson & Johnson, Medical Brasil). After 12 h, fast basal glucose was measured at time 0, and after the administration of glucose (2 g/kg, i.p.), measurements were taken at 10, 20, 30, 45, 60, and 120 min to obtain the glucose curve.

### Metabolic Cage and Biochemical Analysis

The animals were allocated to metabolic cages for 3 days, with the first 24 h for acclimatization and the next 24 h for collection. In order to calculate the food and water consumption, all cages had free access to 40 g of ground feed and 200 mL of water. Furthermore, feces and 24-h urine were collected in cryotubes and stored in a freezer at − 80 °C for further analysis. Immediately after the metabolic cage period, rats were anesthetized as described above to allow blood collection from the thoracic vena cava. Blood was centrifuged (3000 rpm, 10 min) and the serum was separated into cryotubes and stored at − 80 °C. Microalbuminuria, creatinine, urea, and blood urea nitrogen (BUN) were analyzed using the enzymatic kinetic method (Labmax 100 biochemical analyzer; Labtest) according to the manufacturer's instructions.

### Systolic Blood Pressure Recordings

In order to determine baseline systolic blood pressure (SBP), the tail plethysmography method was used according to the manufacturer's instructions (IITC Life Science, 23,924 Victory Blvd, Woodland Hills, CA, USA). The final SBP was measured with the animal anesthetized by direct method (femoral cannulation) before the renal function protocol.

### Renal Function

At the end of the treatment, the animals were anesthetized with sodium pentobarbital (50 mg/kg i.p.) and catheterized as follows: trachea (PE 240) to assist in breathing; femoral artery (PE 50) connected to a transducer (TRI 21; Letica Scientific Instruments, Spain) coupled to a digital system (Powerlab/4SP ML750; ADInstruments, Australia) for continuous blood pressure monitoring; vein (PE 50) connected to a continuous infusion pump for constant infusion of 3% mannitol isotonic saline solution, inulin, and para-aminohippurate (PAH) at a rate of 0.06 mL/min; and bladder (PE 320) for urine collection (Fig. 1B).

After a stabilization period of 30 min, a prime dose of inulin and PAH (30 mg/100 g and 0.6 mg/100 g body weight, respectively) was administered intravenously, then a continuous infusion of these substances began with four periods of 30 min for urine and blood collections. Urinary volume was determined gravimetrically. Hematocrit analysis was carried out with heparinized capillary tubes. Plasma and urinary inulin and PAH concentrations were measured by colorimetric assay. The following parameters were analyzed: glomerular filtration rate (GFR) through inulin clearance and renal plasma flow (RPF) using PAH clearance, where from the determination of these parameters, renal blood flow (RBF = RPF/1 − hematocrit) and renal vascular resistance (RVR = mean arterial pressure/RBF) were obtained. The determination of plasma and urinary inulin values was performed using the modified Fuhr method [49].

### Total Nitrogen Oxides—NOx

The right kidney was homogenized in phosphate buffered solution (PBS 1 × pH 7.4), deproteinized in a 100 µL:200 µL dilution of 100% ethanol, centrifuged at 3000 rpm (4 °C, 10 min), and the supernatant stored. To obtain the standard curve for nitrate, a serial dilution was established with working solution (WS) (200 µM sodium nitrite [NaNO_2_]), vanadium chloride, *N*−1-naphthylethylenediamine dihydrochloride (NEED 1%), and sulfanilamide 1%. For NOx in the kidney dosage, we performed substitution of the WS with the deproteinized homogenate along with the other reagents to avoid generation of total nitrogen oxides [50]. The dosages were performed in duplicate, read at 540 nm (Epoch 2 Microplate Spectrophotometer—BioTek), and expressed in micromolar per milligram of proteins (previously quantified by the Bradford method [51]).

### Evaluation of Oxidative Stress

#### Measurement of Intracellular ROS

The reactive oxygen species (ROS) production was detected by flow cytometry as previously described [52–54]. Intracellular superoxide anion (O_2_^**−**^), hydrogen peroxide (H_2_O_2_), and peroxynitrite (ONOO^−^) levels were analyzed separately by dihydroethidine (DHE), dichlorofluorescein (DCF), and hydroxyphenyl fluorescein (HPF), respectively. Concisely, 1 × 10^6^ cells were incubated with 160 mM/L of DHE, 20 mM/L of DCF, and 5 mM/L of HPF at 37 °C for 30 min in the dark. The cells were then washed and resuspended in PBS cytometry and analyzed by flow cytometry (10,000 events; FACSCanto II, BD). Data were obtained using the FACS Diva software (BD) and overlay histograms analyzed using Flow-Jo software. Data were evaluated through changes in median fluorescence intensity (MFI) and expressed in arbitrary units (a.u.).

#### Advanced Oxidation Protein Products and Thiobarbituric Acid Reactive Substances

The quantification of advanced oxidation protein products (AOPP) was conducted as previously described [55], with some adaptations. For the standard curve, different concentrations of chloramine-T standard solution were added to the microplate, with 10 μL of potassium iodide (KI) (1.16 mM) and 20 μL of acetic acid. Subsequently, the same procedure was performed with the supernatant of the sample (200 µL) previously centrifuged at 11,000 rpm for 20 min (4 °C), and 10 μL of KI and 20 μL of acetic acid were added. After shaking the microplate for 6 min, the reading was performed at 340 nm in the spectrophotometer (Epoch 2 Microplate Spectrophotometer—BioTek).

For the determination of thiobarbituric acid reactive substances (TBARS), lipid peroxidation levels were evaluated according to the method previously described [56]. Then, 250 μL of each homogenate was mixed with 500 μL of reagent solution composed of 0.375% thiobarbituric acid (TBA), 15% trichloroacetic acid, and 0.25 M hydrochloric acid. The mixtures were then placed in a water bath at 90 °C for 15 min. After cooling on ice for 2 min, they were centrifuged at 3000 rpm for 10 min (4 °C), and 200 μL of the supernatant was transferred to a microplate for reading at 532 nm in the spectrophotometer (Epoch 2 Microplate Spectrophotometer—BioTek). A malondialdehyde (MDA) calibration curve (0–25 μM) was used as a standard.

The samples were prior quantified by the Bradford method [51]. Therefore, the AOPP and TBARS results were expressed in chloramine T equivalents per milligram of protein (µM/mg of protein) and MDA per milligram of protein, respectively.

#### Picrosirius Red

The left kidney was fixed in a buffered formaldehyde solution (37% concentration) with 10% saline for 24 h, followed by paraffin embedding in the automated processing equipment (Lupetec PT05). For visualization of collagen deposition, 5-μm-thick sections (Leica RM2125 RTS rotary microtome) were mounted on slides and stained with Picrosirius red. The samples were photographed at × 400 magnification using a Leica DM500 trinocular optical microscope with an ICC50 HD photo documentation system and subsequently quantified using ImageJ software.

### Statistical Analyses

The data were analyzed using one-way ANOVA or two-way ANOVA followed by Fisher's post hoc test. The analyses were performed using GraphPad Prism 8 and presented as mean ± standard error of the mean (SEM); *p* < 0.05 was considered significant, followed by the legend: comparison with group O (*), OF (†), and OK (#).

## Results

### Body Weight, Tissue Weight, Tissue/Tibia Ratio, and SBP

It is possible to observe through the ponderal parameters (Table 2) that both the Δ weight and visceral fat showed a significant increase in the fructose groups (OF and OKF). Regarding the organ weights and their respective ratios (g/cm^2^), there was no difference between the groups in the weights of the kidney and uterus.

**Table 2:** Evaluation of ponderal data, tissue weight, tissue/tibia ratio, and systolic blood pressure in ovariectomized female SHR submitted to high fructose intake, kefir treatment, or high fructose intake plus kefir

	O	OF	OK	OKF
∆ Weight (g)	65.14 ± 3.11	90.29 ± 5.87^*#^	71.43 ± 4.29	95.00 ± 5.28^*#^
Visceral fat (g)	2.86 ± 0.38	5.29 ± 0.93^*#^	2.92 ± 0.41	5.63 ± 0.94^*#^
RK (g)	0.89 ± 0.0216	0.91 ± 0.0417	0.95 ± 0.0364	0.95 ± 0.0636
RK/tibia ratio (g/cm^2^)	0.2568 ± 0.0058	0.2636 ± 0.0130	0.2779 ± 0.0134	0.2750 ± 0.0172
WU (g)	0.0722 ± 0.0024	0.0651 ± 0.0029	0.0798 ± 0.0070	0.0738 ± 0.0077
WU/tibia ratio (g/cm^2^)	0.0206 ± 0.0006	0.0186 ± 0.0008	0.0232 ± 0.0021	0.0212 ± 0.0021
Initial SBP (mmHg)	149.8 ± 3.24	139.5 ± 3.29	143.7 ± 2.45	145.9 ± 3.76
Final SBP (mmHg)	193.2 ± 5.87	184.8 ± 18.40	199.7 ± 3.45	205.1 ± 5.03

Data are presented as mean ± SEM and analyzed by one-way ANOVA followed by Fisher's post hoc test, *n* = 6–7 per group

*RK* right kidney, *SBP* systolic blood pressure, *WU* wet uterus, *O* ovariectomized group, *OF* ovariectomized fructose group, *OK* ovariectomized kefir group, *OKF* ovariectomized kefir + fructose group

*p < 0.05 vs O; †*p* < 0.05 vs OF; #*p* < 0.05 vs OK

Additionally, no significant difference was found between the groups in either the initial systolic blood pressure (SBP) assessed by indirect measurement or in the final SBP by direct measurements. It can be observed that in our experimental model, fructose and kefir did not alter blood pressure.

### Fasting Glucose and Glucose Tolerance Test

**Fig. 2:**
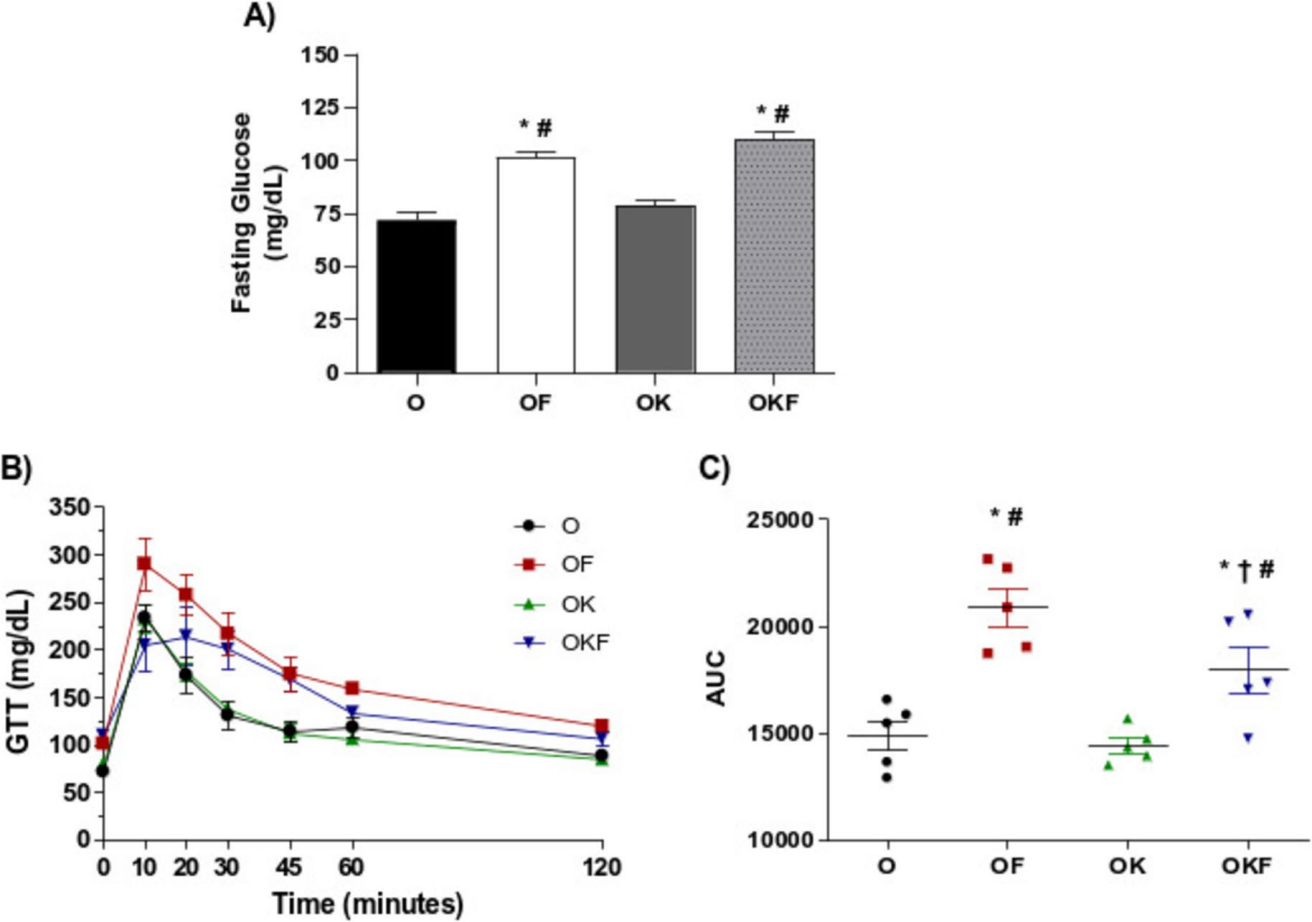
Kefir partially improved glucose intolerance induced by fructose intake. (**A**) Final fasting blood glucose, (**B**) glucose tolerance test (GTT), and (**C**) AUC of GTT. Data are presented as mean ± SEM and analyzed by one-way or two-way ANOVA followed by Fisher's post hoc test, *n* = 5 per group. *p < 0.05 vs O; †*p* < 0.05 vs OF; #*p* < 0.05 vs OK

Both groups exposed to fructose showed an increase in fasting blood glucose (Fig. 2A). In Fig. 2B, we can observe that the fructose groups exhibited glucose intolerance compared to the O and OK groups. The OF group showed a more pronounced curve compared to OKF, which showed a reduction in the peak glucose tolerance response at the 10- and 20-min points on the graph. The effect observed in the OKF group was reinforced by the partial reduction of area under the curve (AUC) (Fig. 2C).

### Metabolic Cage and Biochemical Analyses

**Fig. 3:**
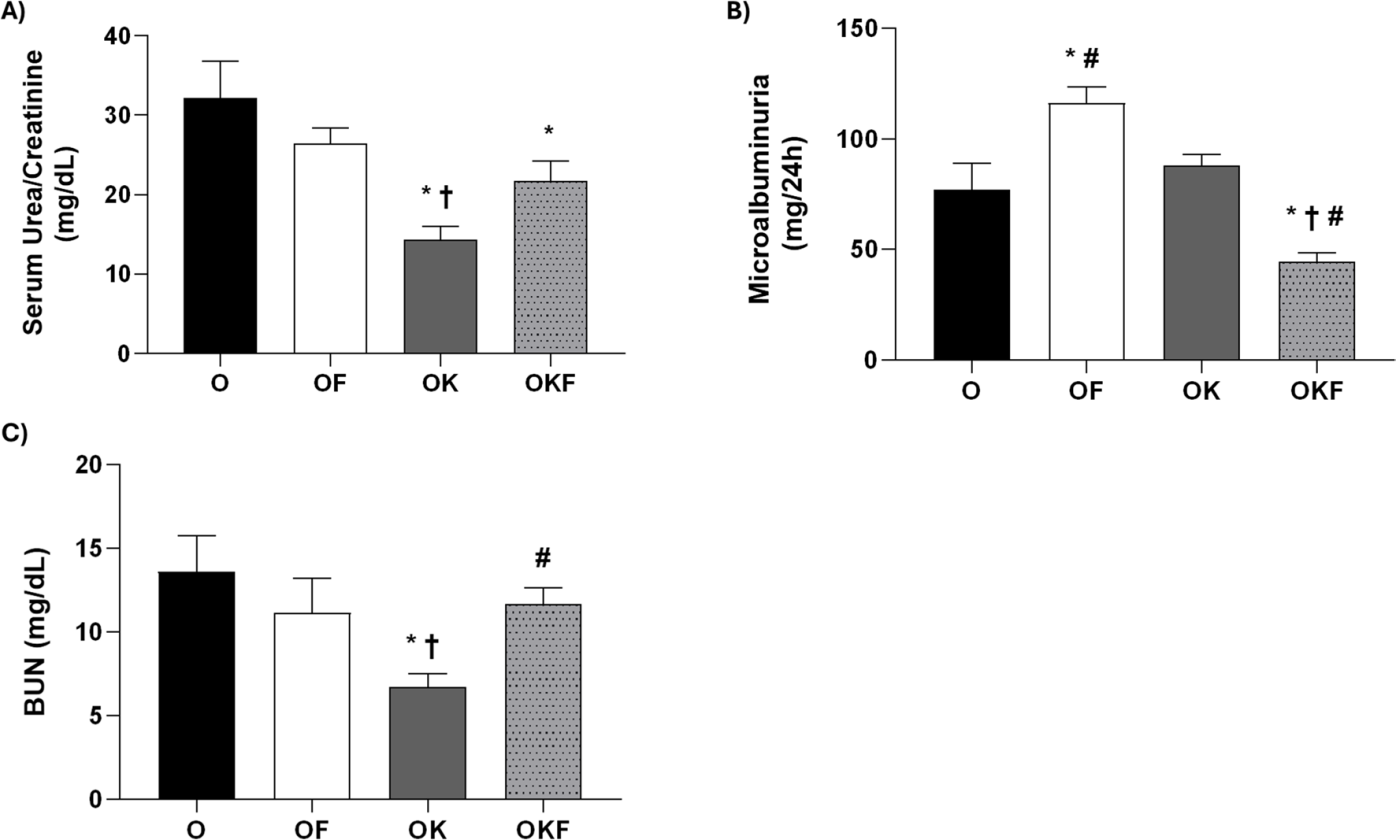
Beneficial effects of kefir on decreased injury markers. (**A**) Microalbuminuria, (**B**) serum urea/creatinine ratio, and (**C**) blood urea nitrogen (BUN). Data are presented as mean ± SEM and analyzed by one-way ANOVA followed by Fisher's post hoc test, *n* = 5–7 per group. *p < 0.05 vs O; †*p* < 0.05 vs OF; #*p* < 0.05 vs OK

No significant difference was found between the groups in water and food consumption, volume of feces and urine, and urinary flow (Table 3). The OK group showed a significant reduction in serum urea and BUN compared to the other groups (Fig. 3C). Moreover, the OF group did not differ from the control group, and the reduction in serum urea caused by kefir disappeared in the OKF group. However, there was no difference between the groups in urinary urea.

**Table 3:** Metabolic cage and biochemical analyses in ovariectomized female SHR submitted to high fructose intake, kefir treatment, or high fructose intake plus kefir

	O	OF	OK	OKF
Metabolic cage				
Water intake (mL)	30.83 ± 3.516	28.13 ± 1.315	32.50 ± 2.113	32.86 ± 2.641
Food intake (g)	16.29 ± 0.9184	18.00 ± 1.069	16.50 ± 1.296	17.71 ± 1.409
Feces (g)	13.43 ± 1.04	12.50 ± 0.7319	13.75 ± 0.7962	10.57 ± 1.360
Urine (mL)	11.75 ± 1.289	11.25 ± 0.4818	12.93 ± 0.6851	12.00 ± 0.9880
Urinary flow (mL/24 h)	0.4896 ± 0.0537	0.4688 ± 0.0200	0.5387 ± 0.0285	0.5000 ± 0.0411
Serum				
SUr (mg/dL)	29.17 ± 4.600	23.88 ± 4.410	14.38 ± 1.711^*†^	25.00 ± 2.082^#^
SCr (mg/dL)	0.9167 ± 0.0749	0.8000 ± 0.0436	1.086 ± 0.0404^*†^	1.167 ± 0.0557^*†^
Urine				
UUr (mg/24 h)	16.492 ± 688.3	22.587 ± 1.870	19.360 ± 1.947	20.100 ± 1.369
UCr (mg/24 h)	633.3 ± 27.15	806.7 ± 32.99^*^	1.482 ± 79.14^*†^	1.573 ± 48.60^*†^

Data are presented as mean ± SEM and analyzed by one-way ANOVA followed by Fisher's post hoc test, *n* = 6–8 per group

*SUr* serum urea, *SCr* serum creatinine, *UUr* urine urea, *UCr* urine creatinine, *O* ovariectomized group, *OF* ovariectomized fructose group, *OK* ovariectomized kefir group, *OFK* ovariectomized kefir + fructose group

*p < 0.05 vs O; †*p* < 0.05 vs OF; #*p* < 0.05 vs OK

Both groups that received kefir showed an increase in creatinine compared to O and OF. In urinary creatinine, the groups that received kefir also showed the same pattern of response, although the OF group also had an increase in this parameter compared to the control group. On the other hand, in terms of the urea/creatinine ratio (Fig. 3B), the OK group showed a reduction compared to the O and OF groups. Additionally, the OKF group also reduced this parameter compared to O, but was not different from OF. In other words, kefir was able to prevent only the increase established by ovariectomy.

In terms of microalbuminuria (Fig. 3A), the group that received fructose overload had a significant increase compared to the other groups. Although kefir did not differ from the control group (O), the probiotic was able to reduce this parameter in the OKF group, which differed from all other groups.

### Renal Function

**Fig. 4:**
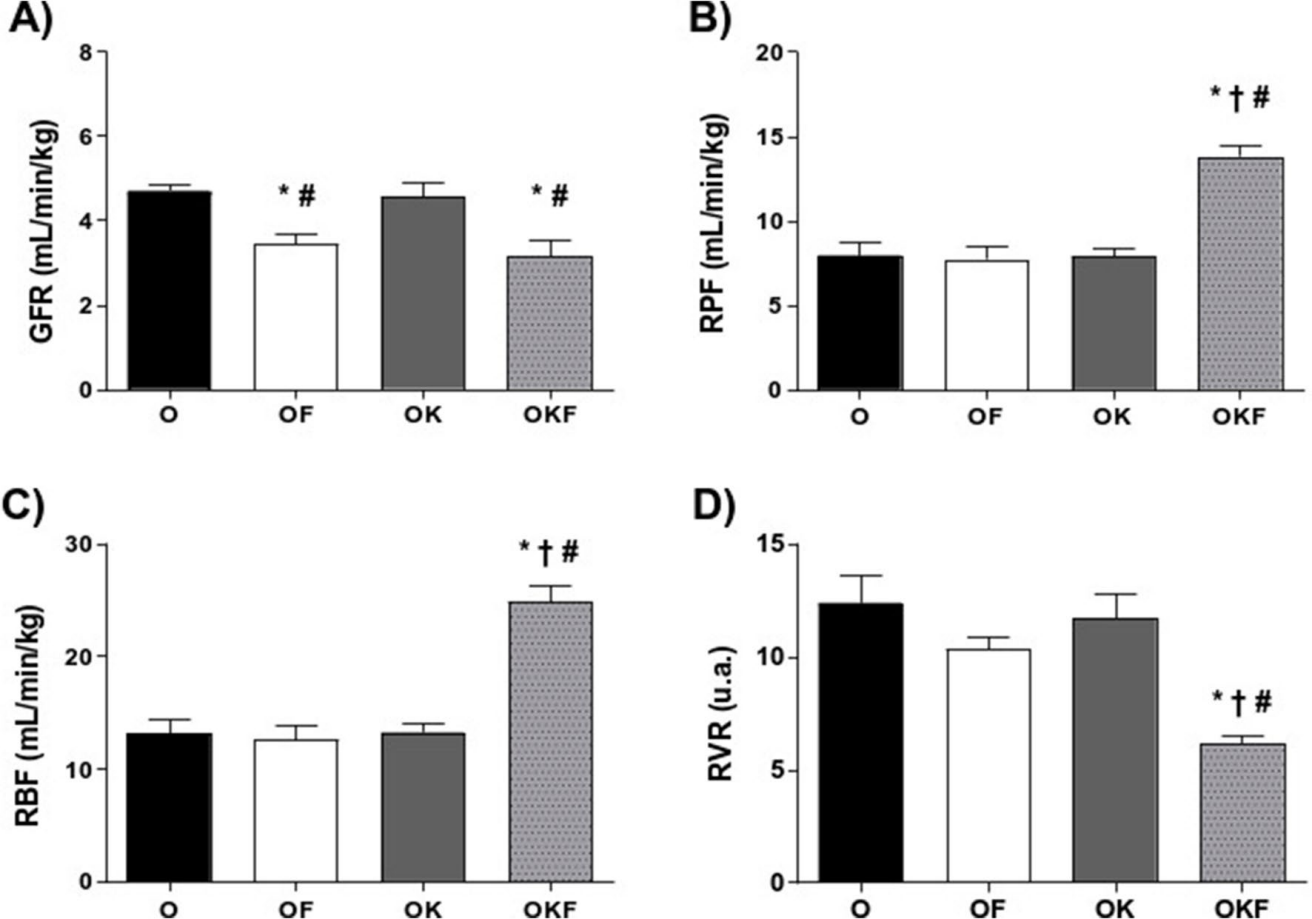
Effects of fructose and kefir on renal function. (**A**) Inulin clearance equivalent to glomerular filtration rate, (**B**) PAH clearance equivalent to renal plasma flow, (**C**) renal blood flow, and (**D**) renal vascular resistance. Data are presented as mean ± SEM and analyzed by one-way ANOVA followed by Fisher's post hoc test, *n* = 5 per group. *p < 0.05 vs O; †*p* < 0.05 vs OF; #*p* < 0.05 vs OK

The results of renal function show that both groups that received fructose showed a reduction in GFR (Fig. 4A). In addition, there was no significant difference between the OK group and the control group, indicating that the kefir probiotic was not able to prevent the damage triggered by fructose.

Regarding RPF (Fig. 4B) and RBF (Fig. 4C), the OF and OK groups did not show a difference when compared to the control group. However, in animals that received preventive treatment with kefir in association with fructose (OKF), there was a significant increase compared to the other groups in both parameters. Furthermore, in the RVR, which is inversely proportional to RBF, there was a significant reduction in OKF group compared to the other groups (Fig. 4D).

### Bioavailability of Oxide Nitric and Oxidative Stress

**Fig. 5:**
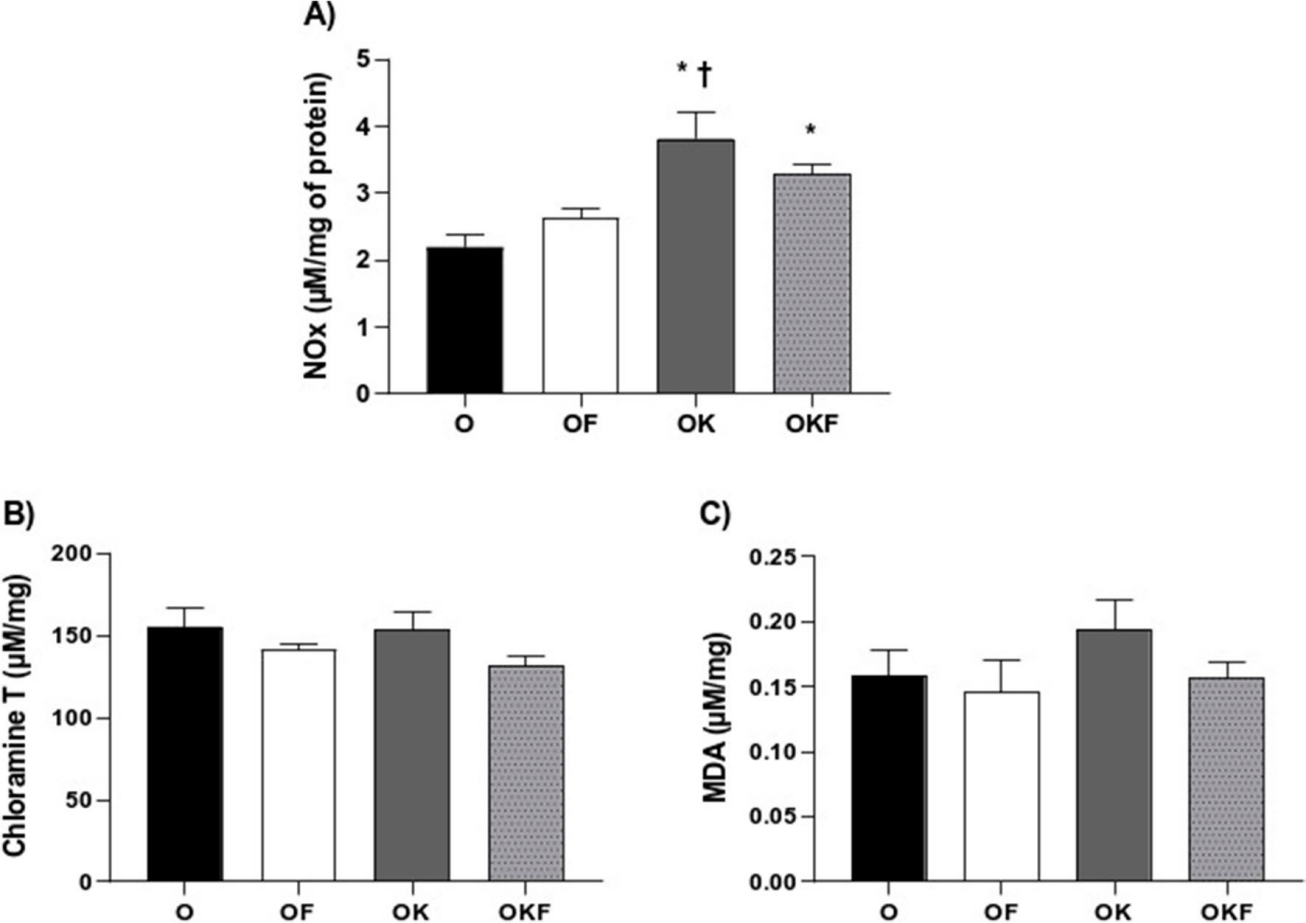
Effects of fructose and kefir in the bioavailability of nitric oxide, protein oxidation, and lipid peroxidation on renal tissue. (**A**) Total nitrogen oxides (NOx), (**B**) chloramine T (advanced oxidation protein products—AOPP), and (**C**) MDA (thiobarbituric acid reactive substances—TBARS). Data are presented as mean ± SEM and analyzed by one-way ANOVA followed by Fisher's post hoc test, *n* = 5–7 per group. *p < 0.05 vs O; †*p* < 0.05 vs OF; #*p* < 0.05 vs OK

Regarding the amount of NOx quantified by the nitrate reduction dosage (Fig. 5A), it can be observed that the OK and OKF groups showed a higher concentration compared to the control group. Furthermore, the OK group also differentiates from OF group.

As to the results of AOPP and TBARS, represented respectively by the chloramine T and MDA graphs (Fig. 5B, C), it can be observed that both parameters did not differ between the groups in renal tissue.

**Fig. 6:**
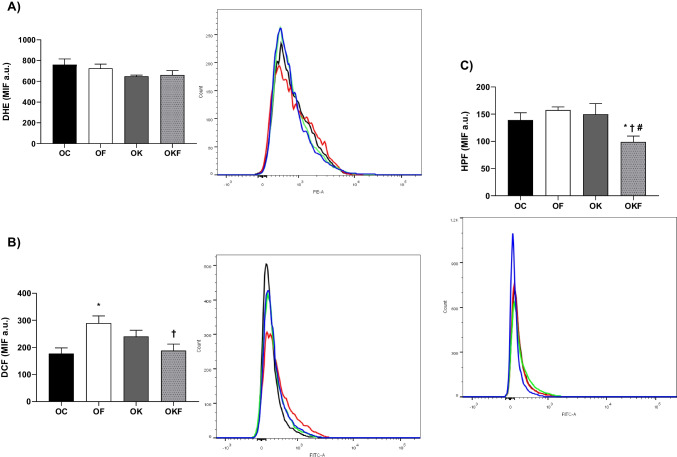
Determination of cytoplasmic levels of superoxide anion (O_2_^−^), hydrogen peroxide (H_2_O_2_), and peroxynitrite (ONOO.^−^) in renal cells. (**A**) Dihydroethidine (DHE), (**B**) dichlorofluorescein (DCF), and (**C**) hydroxyphenyl fluorescein (HPF). Data are presented as mean ± SEM and analyzed by one-way ANOVA followed by Fisher's post hoc test, *n* = 6–7 per group. *p < 0.05 vs O; †*p* < 0.05 vs OF; #*p* < 0.05 vs OK. Additionally, there is a representative figure below each graph with its respective flow cytometry histogram (PE-A reading channel for DHE; FITC-A for DCF and HPF)

On the other hand, in the flow cytometry in renal cells (Fig. 6), it was observed that there was no difference between the groups in O_2_^−^ levels. However, in the levels of H_2_O_2_, the OF group showed a significant increase compared to the control and OKF groups. Additionally, the OKF group showed a reduction in ONOO^−^ compared to the other groups.

### Collagen Deposition

**Fig. 7:**
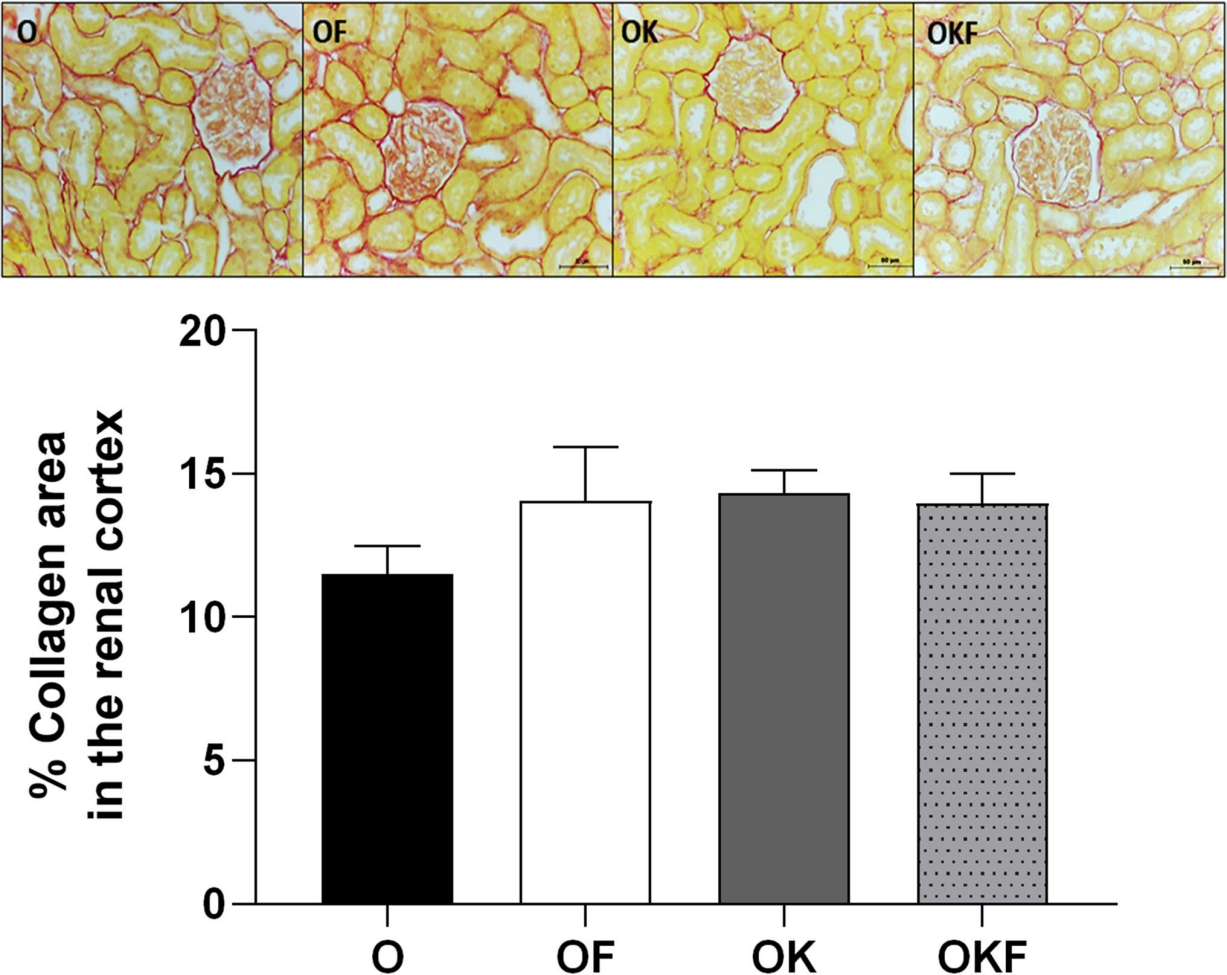
Percentage of collagen area per glomerulus in the renal cortex, with respective Picrosirius red images from each group. Data are presented as mean ± SEM and analyzed by one-way ANOVA, *n* = 5–6 per group

In the analysis of renal remodeling based on collagen deposition in the glomeruli, no difference between the groups was observed (Fig. 7).

## Discussion

In this study, we have shown for the first time that kefir presented favorable effects in a complex model of MetS associated with ovarian hormone deficiency (OKF), possibly protecting the kidney from the deleterious effects of fructose, leading to changes such as partial reduction in glucose intolerance, reduction in microalbuminuria and urea/creatinine ratio, increase in NOx, and reduction in hydrogen peroxide and peroxynitrite.

### Kefir in the OKF Group Partially Improved Glucose Intolerance

Our results demonstrated that fructose-overloaded groups (OF and OKF) showed increased weight, visceral fat, and fasting blood glucose (Table 2, Fig. 2), as well documented in the literature [11, 14, 57]. Our data corroborate the results of Majumdar et al., who showed that in an animal model of menopause, fructose appears to exacerbate the effects of ovarian deficiency. Several studies have been conducted in humans and animals describing that fructose promotes dyslipidemia, increased visceral adiposity, lipid accumulation in the liver and kidney, and insulin resistance [58–61]. On the other hand, in our experimental model, kefir did not prevent weight gain and abdominal fat deposition. Similar data were observed in an OVX mouse model, in which the OVX group treated with kefir did not differ in weight gain [62]. However, in other experimental models, a reduction in lipid profile, weight gain, and abdominal fat mass has been observed through mechanisms attributed to kefir in reducing the gene expression of adipogenesis and lipogenesis [62–66].

Nevertheless, kefir did not alter blood glucose in the OK and OKF groups, and it was able to partially improve glucose intolerance in the OKF. Studies indicate that kefir improves glucose tolerance and reduces insulin resistance in a non-alcoholic fatty liver disease model in male mice [67], and it was able to promote a reduction in blood glucose and partially reduce GTT when compared to the untreated diabetic group [68, 69]. It is suggested that fructose overload leads to alterations in the gut microbiome and increased permeability of the intestinal wall [70–72]. Furthermore, Urdaneta et al. described that in the intestine, kefir modulates the substrate affinity and activity of dipeptidyl peptidase IV enzyme, responsible for glucose homeostasis and is involved in the rapid metabolism of glucagon [73].

### Kefir Showed Positive Results on Kidney Injury Marker

In the present study, along with the washout period, it likely led to no differences between the groups in the metabolic cage parameters (Table 3). However, following the biochemical analyses, it can be observed that although there was no difference between the groups in the urinary excretion of urea, the OK group was able to reduce the serum urea. Meanwhile, in serum and urinary creatinine, both kefir-treated groups showed an increase when compared to the others.

A study conducted with female Wistar rats observed that the OVX group did not show a difference in plasma urea and creatinine compared to the control group. However, the diabetic OVX rats had a significant increase in both parameters [74]. On the other hand, Seraphim et al. did not observe a difference in serum urea and creatinine between female Wistar rats that received water and those that received fructose, similar to our result obtained in serum [75].

Kahraman et al. demonstrated an increase of serum creatinine and urea, microalbuminuria, and a reduction of urinary creatinine in diabetic male Sprague–Dawley rats, whereas the treatment with kefir was able to revert these alterations and increased the urinary excretion of creatinine [76]. Furthermore, in an ischemia reperfusion model, the group treated with kefir (1 mL/100 g) for 30 days showed a reduction in serum urea and creatinine [77]. However, in diabetic male Wistar rats treated with kefir (1.8 mL/day) for 8 weeks, a reduction in plasma urea was observed, while urinary urea increased. In addition, kefir showed no difference in the urinary and plasma creatinine [69]. Therefore, these studies suggest that the differences found in urea and creatinine may be related to the treatment dose and the different animal models studied, which in turn present different pathophysiologies, as well as the female SHR model of the present study. As previously mentioned, the urinary excretion of these metabolites is important for homeostatic control, where kefir showed positive results.

Moreover, we speculate that there may be a relationship between the increase in serum creatinine and treatment with the probiotic kefir, which in turn is rich in proteins [36, 78]. It is well known that proteins ingested in the diet produce creatine phosphate by the liver, and its consumption by the muscle generates energy, ultimately producing creatinine that is subsequently eliminated by the kidneys. It is believed that this increase in serum creatinine promoted by kefir treatment is not a negative aspect due to the urea/creatinine ratio. Considering that the increase in urea/creatinine ratio is indicative of impairment of renal function [79, 80], kefir effectively reduced this ratio in the O group.

Another marker of renal dysfunction that was evaluated is BUN, in which kefir reduced when compared to the ovariectomy group, but it was not able to improve in the OKF group (Fig. 3). Studies have shown that BUN is strongly associated with acute kidney injury and chronic kidney disease [81–84]. Here, we demonstrated that kefir is able to protect the kidney in situations of hypertension and ovarian deficiency.

### Kefir Prevented the Increase of Microalbuminuria Caused by Fructose

As observed in our results, the OF group showed an increase of microalbuminuria compared to the other groups. Therefore, the increase in this parameter, combined with the reduction in GFR in the OF group, may indicate renal insufficiency. Our data are corroborated by the literature, which demonstrates that the severity of renal function impairment manifests through reduced GFR and increased microalbuminuria, indicating renal excretory function and dysfunction in the glomerular barrier, respectively [85]. In certain renal diseases, negative charges on the membranes of glomerular epithelial cells can be lost even before significant histological changes occur. Additionally, studies indicate that fructose consumption leads to microalbuminuria [86, 87].

Furthermore, although the OK group did not show a difference from the control group (O), the probiotic reduced microalbuminuria in the OKF group, which differed from all other groups. Interestingly, while the OKF group also showed a reduction in GFR like OF group, kefir seems to have acted protectively on the glomerular epithelial cell membranes, reducing the excessive excretion of microalbumin caused by fructose.

Regarding renal function, several studies in male and female SHR describe that this model presents a primary natriuretic defect of the angiotensin II receptor type 2 in proximal tubule cells, increased sodium reabsorption in the proximal tubule, and increased activity of sodium–hydrogen exchanger 3, sodium/glucose cotransporter 2, and sodium/hydrogen ATPase pump, resulting consequently in increased fluid and sodium reabsorption, contributing to the establishment of hypertension in this lineage [88–91]. Additionally, it is well described in the literature that female sham SHR present superior renal function parameters compared to the OVX group due to the presence of postulated protective female hormones [18, 19, 92, 93]. In a study conducted by our research group, it was possible to observe that castrated female SHR when compared to the control group (sham) presented a significant impairment in GFR and RPF. Additionally, ovariectomy resulted in a significant decrease in angiotensin converser enzyme 2 activity, which may have reduced the synthesis of angiotensin 1–7. These changes in the non-classical pathway of the renin–angiotensin system may have contributed to the renal dysfunction in OVX rats [18]. Therefore, OVX SHR, which were our control group (O), already presented alterations in renal function, which were further impaired by fructose.

### Kefir Increased NOx and Blood Flow in the OKF Kidney

As previously described, fructose has several mechanisms capable of modulating the renin–angiotensin system and sympathetic system and increasing angiotensin II, in addition to reducing the bioavailability of nitric oxide (NO) through increased uric acid, which triggers increased vasoconstrictor responses [11, 15, 94]. Moreover, fructose consumption induces glomerular hypertension and sclerosis in the afferent arteriole [95]. However, in the OKF group the treatment with kefir was able to increase RPF and RBF and reduce RVR. This increase in blood flow may be due to greater formation and/or bioavailability of nitric oxide (NOx) in renal tissue from the OKF group. On the other hand, when observing the group treated only with kefir, although it also increased the bioavailability of NOx, it presented the same response pattern as the control group. We can propose that in OKF, the increase in NO and reduction of peroxynitrite could lead to a reduction in the tone, counteracting the vasoconstrictor effects of fructose, resulting in the alterations observed in the renal flow of the OKF group. Additionally, we cannot rule out the possibility that nitrite itself may have a vasodilatory effect, as previously demonstrated [96].

Despite this vasodilatory effect in the kidney, no reduction in systemic blood pressure was observed in our study. The increase in vasodilatory response in the renal vasculature may not be directly related to blood pressure [97]. Besides that, it is possible to observe that both OK and OKF groups had increased NOx in the kidney tissue, suggesting that the probiotic kefir increased the formation of nitrate and nitrite, indicating a greater local formation of NO.

Considering the effects of the fructose exposure and/or the treatment with kefir, the studies showed controversial results, which could be explained by the differences in experimental protocols. Similar to our model, although treated for 19 weeks (100 mg/mL), the authors found that fructose reduced plasma NOx when compared to the control [57]. Meanwhile, in diabetic rats, elevated levels of NO in the renal cortex were found compared to the control group; otherwise, the group treated with kefir showed a reduction in this parameter. Moreover, in the analysis of protein expression in the renal cortex, kefir also showed a reduction in inducible nitric oxide synthase in the diabetic group [68]. Thus, the increase in NO in this condition appears to occur in situations of inflammation.

Cosenzi et al. observed in Wistar Kyoto rats that a fructose-rich diet for 4 weeks resulted in glomerular hypertrophy, increased inducible nitric oxide synthase, and high urinary nitrite/nitrate excretion [98]. This suggests that NO may play an important role in the pathogenesis of renal damage, counteracting the effects promoted by a fructose-rich diet. In studies comparing healthy and diabetic rats, administration of oxide nitric synthase inhibitor eliminated differences in renal hemodynamics between the groups, suggesting that the NO pathway is responsible for the initial changes in hemodynamics [99–102].

Studies indicate that in hypertension and renal disease in non-diabetic rats, the function of neuronal oxide nitric synthase in the macula densa is compromised, where chronic inhibition plays a role in increasing sensitivity of tubuloglomerular feedback, consequently reducing GFR as observed in our experimental model, with fructose as an aggravating factor [103–105]. Consequently, in addition to the time of exposure to the deleterious effects of fructose, hypertension, and the deficiency of sex hormones, more studies are needed to understand the mechanisms and pathways involved in the compensatory process of increased nitric oxide bioavailability.

### Kefir Attenuated the Generation of Hydrogen Peroxide (DCF) and Peroxynitrite (HPF) in the OKF Renal Tissue

Following the results about intracellular ROS (Fig. 6), we observed that there was no difference between the groups in O_2_^−^ levels, but the levels of hydrogen peroxide in the OF group showed a significant increase compared to the control and OKF groups. Additionally, the OKF group showed a reduction in ONOO^−^ compared to the other groups. A previous study conducted by our research group, comparing female SHR sham and OVX, was able to show that castration led to an increase in O_2_^**−**^ and H_2_O_2_ in renal tissue [18]. On the other hand, in another previous study in male Wistar rats that received fructose overload, no difference was found in DHE in the mesenteric artery compared to the control group [14]. Although our control group probably has an increase in O_2_^**−**^, there is no difference between the other groups. Nevertheless, Punaro et al. showed a significant increase in superoxide anion in the renal cortex of diabetic male Wistar rats, which was reduced by kefir [69].

Furthermore, we suggest that the increase in H_2_O_2_ in the OF is associated with the fructose metabolism pathway involving xanthine oxidase, which highlighted that in inflammation, ischemia, and low pH conditions, this pathway serves as an abundant source of ROS, mainly generating H_2_O_2_ [13, 106]. In addition, as a result of the antioxidant benefits from kefir, which is also described to contribute to the increase in superoxide dismutase expression [107], we observe that kefir was capable of attenuating the increase of H_2_O_2_ caused by fructose.

Meanwhile, studies with probiotic kefir showed that male SHR at 4 months of age had increased O_2_^**−**^, H_2_O_2_, and ONOO^−^ levels in the aorta, while the group treated with kefir showed reductions in all these parameters. Additionally, it was also observed that the group treated with kefir had a higher production of NO compared to the other groups, as measured by the 4,5-diaminofluorescein-2/diacetate (DAF) marker [108, 109]. Following the next result of our study, there is no difference between OF and OK groups compared to the ovariectomized SHR group regarding reactive nitrogen species (peroxynitrite). However, kefir reduced this parameter in OKF. On the whole, when observing H_2_O_2_, ONOO^−^, and NOx results of the OKF group, it is suggested that in this group, NO may bind less to O_2_^−^ to form ONOO, thus presenting greater bioavailability of NO in renal tissue, which may have contributed to the results found in renal function (Fig.), showing increased flow and reduced RVR.

### Kefir or Fructose Overload Did Not Alter AOPP, TBARS, and Collagen in the Renal Tissue

No differences were observed between the groups in AOPP and TBARS levels in renal tissue (Fig. 5), which may indicate that the damage from high fructose intake is tissue specific. In addition, according to Yener et al., lipid peroxidation is the breakdown of fatty acids in the cell membrane by free radicals [77]. Therefore, the lack of difference between the groups in the assessment of lipid peroxidation and protein oxidation in renal tissue, combined with the O_2_^**−**^ results (which showed no difference between the groups), may indicate that fructose did not cause damage beyond what is established by ovariectomy, and kefir was not able to prevent this parameter in renal tissue.

Simultaneously, the literature describes that fructose consumption accelerates the progression of chronic kidney disease in female rats [110] and induced MetS leads to renal hypertrophy, afferent arteriolaropathy, glomerular hypertension, and renal vasoconstriction [95], and kefir can reduce the dilation of the glomerular spacing in renal tissue and improve tubular and epithelial structures [76]. However, although changes in renal hemodynamics and NOx levels were observed in the present study, there were no changes in O_2_^**−**^, protein oxidation, lipid peroxidation, or collagen deposition in the kidneys. It is suggested that the dose and duration of treatment were not potentially able to alter the renal morphological structure already established by ovariectomy (Fig. 7) [18].

In brief, looking at the results between the OK and OKF groups for future perspectives of the study, we speculate that the combination of kefir treatment via gavage along with free access to fructose diluted in drinking water may have led to an increase in secondary metabolites from the probiotic itself in the gastrointestinal tract, thus presenting the particularity found in the results of the OKF group. This hypothesis is reinforced by the study by Larosa et al. which demonstrated that replacing sucrose with unconventional sugars in the preparation of sheep's milk kefir improved antagonistic activity against pathogens, the fatty acid profile, and the functionality of the products, which presented higher antioxidant activity, ACE and α-amylase inhibitory activities, and antiproliferative activities [111]. Therefore, due to the insufficiency of studies in experimental models in female SHR, and especially studies with ovarian hormone deficiency, more studies are needed to understand the different mechanisms and results found with the use of this probiotic.

In conclusion, kefir presented favorable effects in the model of metabolic syndrome and ovarian hormone deficiency (OKF), potentially protecting the kidney against the deleterious effects of high fructose intake, improving oxidative stress. Thus, the probiotic kefir may act as an adjuvant for preventive treatment in clinical situations that resemble the studied model.

## Data Availability

No datasets were generated or analysed during the current study.
